# Isovolemic hemodilution in chronic mountain sickness acutely worsens nocturnal oxygenation and sleep apnea severity

**DOI:** 10.5664/jcsm.10136

**Published:** 2022-10-01

**Authors:** Ana Sanchez-Azofra, Francisco C. Villafuerte, Pamela N. DeYoung, Dillon Gilbertson, Wanjun Gu, Esteban A. Moya, Gustavo Vizcardo-Galindo, Rómulo Figueroa-Mujíca, Cecilia Anza-Ramirez, Jose L. Macarlupú, Luu V. Pham, Peter Wagner, Atul Malhotra, Tatum S. Simonson, Omar A. Mesarwi

**Affiliations:** 1 Division of Pulmonary, Critical Care, & Sleep Medicine and Physiology, Department of Medicine, University of California, San Diego, California; 2 Servicio de Neumología, Hospital Universitario de la Princesa, Universidad Autónoma de Madrid, Madrid, España; 3 Laboratorio de Fisiología Comparada, Facultad de Ciencias y Filosofía, Universidad Peruana Cayetano Heredia, Lima, Perú; 4 Division of Pulmonary and Critical Care Medicine, Johns Hopkins University, Baltimore, Maryland; 5 Center for Physiological Genomics of Low Oxygen, University of California, San Diego, California

**Keywords:** chronic mountain sickness, nocturnal oxygenation, phlebotomy, Andean highlanders, high altitude, hypoxia

## Abstract

**Study Objectives:**

Chronic mountain sickness (CMS) is commonly observed among Andean and other highland populations. Sleep-disordered breathing (SDB) is highly prevalent at high altitude, and SDB and nocturnal hypoxemia have been observed in CMS. Phlebotomy is commonly performed to treat CMS, but it is unknown whether reducing hematocrit improves SDB. We hypothesized that isovolemic hemodilution (IVHD) in CMS would reduce SBD severity and improve sleep efficiency.

**Methods:**

Six participants with CMS and 8 without CMS, all residents of Cerro de Pasco, Peru (altitude 4340 m), completed baseline nocturnal sleep studies. CMS participants then underwent IVHD, and nocturnal sleep studies were repeated 24–48 hours after IVHD. We analyzed sleep apnea severity, nocturnal oxygenation, and sleep quality in those with CMS relative to those without CMS, and the effects of IVHD in CMS participants.

**Results:**

Participants with CMS did not have altered sleep architecture, sleep apnea severity, or nocturnal oxygenation relative to non-CMS participants. However, IVHD in CMS increased apnea-hypopnea index (40.9 ± 6.9 events/h to 61.5 ± 7.7 events/h, *P* = .009). IVHD increased oxyhemoglobin desaturation index (*P* = .008) and the percentage of sleep time spent with oxyhemoglobin saturation at or below 80% (*P* = .012). There was no effect of IVHD on sleep efficiency, arousal index, or sleep staging.

**Conclusions:**

In this cohort, CMS was not associated with worsened SDB or changes in sleep architecture. IVHD, a putative therapeutic option for participants with CMS, appears to worsen nocturnal oxygenation and SDB within 48 hours post-IVHD.

## Introduction

Half a billion people worldwide live at moderately high altitude (> 1,500 m), of whom more than 80 million live at altitudes greater than 2,500 m.^1^ Atmospheric pressure decreases with increasing elevation, resulting in a reduction in the partial pressure of oxygen. Hypoxia at high altitude may cause major physiological stress, resulting in increased hemoglobin concentration, changes to ventilation, and increased pulmonary arterial pressure.^2–7^ Nonetheless, populations have lived in high altitude regions for hundreds of generations and exhibit adaptations and maladaptations to this environmental stress.^8^

Chronic mountain sickness (CMS) is a significant source of morbidity in people exposed to chronic hypobaric hypoxia,^9–12^ especially among Andean highlanders, in whom the prevalence of CMS is as high as 10%.^13^ Its prevalence increases with higher altitude, age, and menopause. Above 4,000 m in the central Andes of Peru, > 30% of highlanders by their mid-50s, and 77% of postmenopausal women, experience CMS.^14–17^ CMS is characterized by excessive erythrocytosis (hemoglobin concentration > 19 g/dL in females, > 21 g/dL in males). Some CMS patients present with severe hypoxemia and/or pulmonary hypertension, which may evolve to frank right heart failure (*cor pulmonale*).^18^ CMS was first described by Monge et al in 1928^19^; the current diagnosis of CMS is determined using the Qinghai Score, which is based both on hemoglobin concentration (the presence or absence of excessive erythrocytosis) and the presence and extent of the following signs and symptoms: breathlessness and/or palpitations, sleep disturbance, cyanosis, venous dilatation, paresthesia, headaches, and tinnitus.^18^ CMS symptoms gradually resolve with descent to lower altitudes and recur after returning to high altitude. Thus, traveling to lower altitude is only a temporary solution for those with CMS. Hyperviscosity from polycythemia is thought to be a major cause of CMS complications. As such, phlebotomy (blood removal without volume replacement) is commonly used to treat CMS symptoms.^20^ Some studies suggest that phlebotomy or hemodilution (blood removal with volume replacement), each of which decreases hematocrit, improves oxygenation and reduces CMS symptoms.^21–24^

Sleep is a vulnerable period in terms of physiological stress, during which hypoxemia may worsen. The prevalence of sleep-disordered breathing (SDB) is greatly increased among highlanders. One recent study in Andeans at high altitude demonstrated a 2-fold increase in apnea-hypopnea index (AHI) relative to those at sea level, driven primarily by an increase in central sleep apnea.^25^ Excessive erythrocytosis is associated with more severe sleep apnea and decreased oxyhemoglobin saturation during sleep in men and women.^26–33^ This relationship may be bidirectional, as sleep apnea is a known risk factor for CMS as well.^18^ In CMS patients, phlebotomy is anecdotally associated with improved sleep, but the effects of phlebotomy or hemodilution on sleep and SDB have not been studied systematically. Therefore, we aimed to characterize SDB severity and sleep quality in patients with CMS and in non-CMS controls, and to evaluate the impact of isovolemic hemodilution (IVHD) on measures of sleep quality and SDB severity in CMS participants. Given the association between elevated hematocrit in CMS and poor sleep quality and SDB, we hypothesized that IVHD in CMS participants would improve sleep efficiency and sleep apnea severity.

## Methods

### Ethical declaration

This study was approved by the University of California, San Diego Human Research Protection Program (UC San Diego Project #171772) and the Institutional Ethics Committee of Universidad Peruana Cayetano Heredia (CIEH-UPCH 081-03-17, SIDISI #59285). Written informed consent was obtained from each participant upon receiving a detailed description of the study protocol in Spanish.

### Study protocol

All study procedures were performed at the Instituto de Investigaciones de la Altura of the Universidad Peruana Cayetano Heredia in the city of Cerro de Pasco, Peru (4,340 m). During a first visit, each participant was interviewed to obtain detailed information regarding medical history in order to exclude patients who did not meet inclusion criteria. In this visit, capillary blood samples were collected to assess hematocrit levels.

During a second visit, a venous blood sample was collected, blood volume was measured, and cardiac output was assessed. On the night of the second visit, sleep studies were performed. During the next 2 days, IVHD was completed in the CMS participants. Forty-eight hours after IVHD, blood volume and cardiac output measurements and sleep studies were repeated. This protocol is part of a larger study investigating the impact of IVHD on exercise physiology in participants with CMS.

### Participant recruitment and baseline screening

The study involved 14 male volunteers between the ages of 24 and 64, 8 without CMS and 5 with CMS (defined as a total Qinghai Score ≥ 6); a sixth participant (age 24) with excessive erythrocytosis (hemoglobin 23.5 g/dL) and intermittent but not constant headaches was also included in the CMS group despite a CMS score < 6, due to the high likelihood that his symptoms were not explicable by another condition; as such, this participant underwent the IVHD protocol. All participants were born above 4,000 m, and were lifelong residents of Cerro de Pasco, Peru (altitude 4,340 m). Participants with preexisting cardiovascular, pulmonary, or kidney disease were excluded from the study, as these conditions may cause secondary CMS.^9^ Participants with abnormal electrocardiogram (Quark C123; Cosmed, Albano Laziale, Italy) or spirometry (Pony FX; Cosmed), (forced vital capacity [FVC] or forced expiratory volume in 1 second [FEV_1_] < 80% predicted value, or an FEV1/FVC ratio < 70%)^34^ were also excluded, as were current smokers, those with a history of employment in mining, anyone who had undergone blood transfusions or phlebotomies in the preceding 6 months, or those who had traveled to lower altitudes (< 3,000 m) for more than 7 days in the preceding 6 months. Heart rate and peripheral oxyhemoglobin saturation (SpO_2_) were measured by pulse oximetry (Nellcor *N*-560 oximeter; Nellcor Puritan Bennet Inc., Pleasanton, CA), and blood pressure was measured with a validated oscillometric device (UA-767 Plus; A&D, Japan).

Also at the baseline visit, participants were screened for the presence or absence of CMS, as well as its severity in affected individuals. This was assessed by the Qinghai score, where CMS was defined as a score ≥ 6. The Qinghai Score is based on the occurrence of excessive erythrocytosis ([Hb] > 21 g/dL), and the presence and severity of the following signs and symptoms: headache, shortness of breath or palpitations, sleep disturbance, paresthesia, cyanosis, dilated veins, and tinnitus.^18^ At this screening visit, hemoglobin concentration was determined from microcentrifuged blood samples obtained from a fingertip blood draw, measured in duplicate.

### Blood samples

On the morning after the screening session, fasting peripheral venous blood samples were collected in each participant in order to confirm the presence of excessive erythrocytosis ([Hb] > 21 g/dL) and to measure hemodilution parameters in those with CMS. In each participant in whom excessive erythrocytosis was diagnosed based on a capillary blood draw, this was confirmed by venous sampling. Second sets of venous and arterial blood samples were obtained in CMS participants 48 hours after IVHD for the same parameters. Arterial blood gas samples were obtained in participants with and without CMS concurrent with venous samples.

### Blood volume measurement

Blood volume was determined based on the initial venous blood samples in all CMS participants and again 48 hours after IVHD. We used the indocyanine green (CardioGreen; Sigma-Aldrich, Burlington, MA) dye dilution method.^35–37^ Briefly, a catheter was placed in the cephalic vein with 2 three-way–connected valves. To avoid dye contamination, 1 valve was used for dye injection (0.05 mg/kg), and the second for blood sample collection just prior to, and 5, 10, 15, and 20 minutes after dye injection. Each sample was centrifuged, plasma collected, and its absorbance read at 805 nm. A 4-point calibration curve was obtained by adding increasing indocyanine green amounts to the plasma samples of each participant to obtain final concentrations of 0.85–3.30 mg/ml. Plasma volume was calculated by extrapolating absorbance values to time zero.

### Isovolemic hemodilution

IVHD was performed with the objective of reducing the hematocrit of participants with CMS to 80% of its initial value. We used the methodology of Gross^38^ to calculate the total blood volume to be removed in order to obtain the targeted reduction in hematocrit, based on the formula:

$$V=\frac{2BV\left({\text{Hct}}_{0}-{\text{Hct}}_{\text{F}}\right)}{\left({\text{Hct}}_{0}+{\text{Hct}}_{\text{F}}\right)}$$

where V is the blood volume to be removed, BV is the baseline total blood volume, Hct_0_ is the baseline hematocrit, and Hct_F_ is the target hematocrit after IVHD.

In 2 consecutive days, 450–900 mL of blood was drawn from each CMS participant to reach the target hematocrit value. The volume of blood drawn each day was replaced by the same volume of colloid plasma expander (poligelyne 3.5%, Hisocel). To ensure gradual hemodynamic changes, each hemodilution session was scheduled for 3 hours.

### Sleep studies

Sleep studies were carried out in all 14 participants. Sleep studies were performed in Cerro de Pasco (4340 m) at baseline (24 hours prior to IVHD) for all participants and repeated 48 hours after IVHD for those with CMS. A limited channel polysomnogram (Alice PDx; Philips Respironics, Murrysville, PA) was placed on each participant by a trained technician. This device measured nasal airflow, finger pulse oximetry, thoracic and abdominal excursion, electrooculogram, 2-channel electroencephalogram, and chin electromyogram. In addition, each participant also was fitted with a WatchPAT ONE device (Itamar Medical, Caesarea, Israel), consisting of fingertip peripheral arterial tonometry and pulse oximetry, in order to ensure robust oximetry measurements and to compare output between devices. Studies were scored by a registered polysomnographic sleep technologist in a blinded manner. Apneas and hypopneas were scored using the American Academy of Sleep Medicine Task Force (2017) diagnostic criteria.^39^ The apnea-hypopnea index, a principal measure of the severity of SDB, is defined as the total number of apneas (complete cessations of respiratory flow) and hypopneas (reductions in respiratory flow) per hour of sleep. For CMS participants, we also evaluated the severity of SDB in rapid eye movement (REM) and non–rapid eye movement (NREM) sleep. Obstructive and central hypopneas were discriminated based on optional American Academy of Sleep Medicine criteria. Circulation time was calculated from the mean value of 10 events per study from the PDx device. Sleep quality was assessed based on sleep efficiency, arousal index, and sleep staging, based on measures from the PDx device. The circulation time was determined for each event as the time between resumption of respiratory flow after an apnea and the nadir saturation corresponding to that apnea.

### Cardiac output measurement (PhysioFlow)

We performed impedance cardiography (Enduro; PhysioFlow, Paris, France) to measure cardiac output (Q_T_) during wakeful rest for all participants the same day as the first blood sample. This procedure was repeated in the CMS group 48 hours after hemodilution. Q_T_ measures were normalized to body weight.

### Statistical analysis

Unpaired *t* tests were conducted to reveal differences between the CMS and control participants. Paired Student’s *t* tests were used to find differences in sleep parameters between participants with CMS pre- and posthemodilution. One-way analysis of variance was used to compare proportions of different sleep stages (REM, N1, N2, N3) between control participants and CMS participants, and CMS participants pre- and posthemodilution. Bland-Altman plots were generated to measure the discrepancy in measurements between the PDx and WatchPAT devices.^40^ For each variable of interest, the difference between the measurements using 2 devices (δ) was plotted against the average of the 2 measurements (μ). A linear model δ = β_0_ + β_1_ μ was used to find underlying trends between the differences and means, where β_1_ indicates the trend and β_0_ indicates the offset between the 2 devices. A *P* < .05 was used to establish statistical significance. Data are reported as mean ± standard error of the mean unless otherwise stated.

## Results

### Biometric and hematological measurements

There was no difference in age, weight, and body mass index (BMI) between participants with and without CMS (Table 1). However, IVHD in CMS participants resulted in a reduction of weight and BMI. As expected, the CMS group had higher baseline hemoglobin than non-CMS group (22.2 ± 0.6 g/dL vs 17.8 ± 0.5 g/dL, *P* < .001), and hemodilution effectively decreased hemoglobin (posthemodilution: 17.7 ± 0.2 g/dL, *P* < .001) compared to baseline values. Accordingly, the high CMS scores in the CMS group at baseline (10.0 ± 5.5) were reduced after IVHD (1.2 ± 1.2; *P* = .016) to values similar to those observed in participants without CMS (Table 1).

### IVHD worsens sleep-disordered breathing in CMS participants

**Figure 1:**
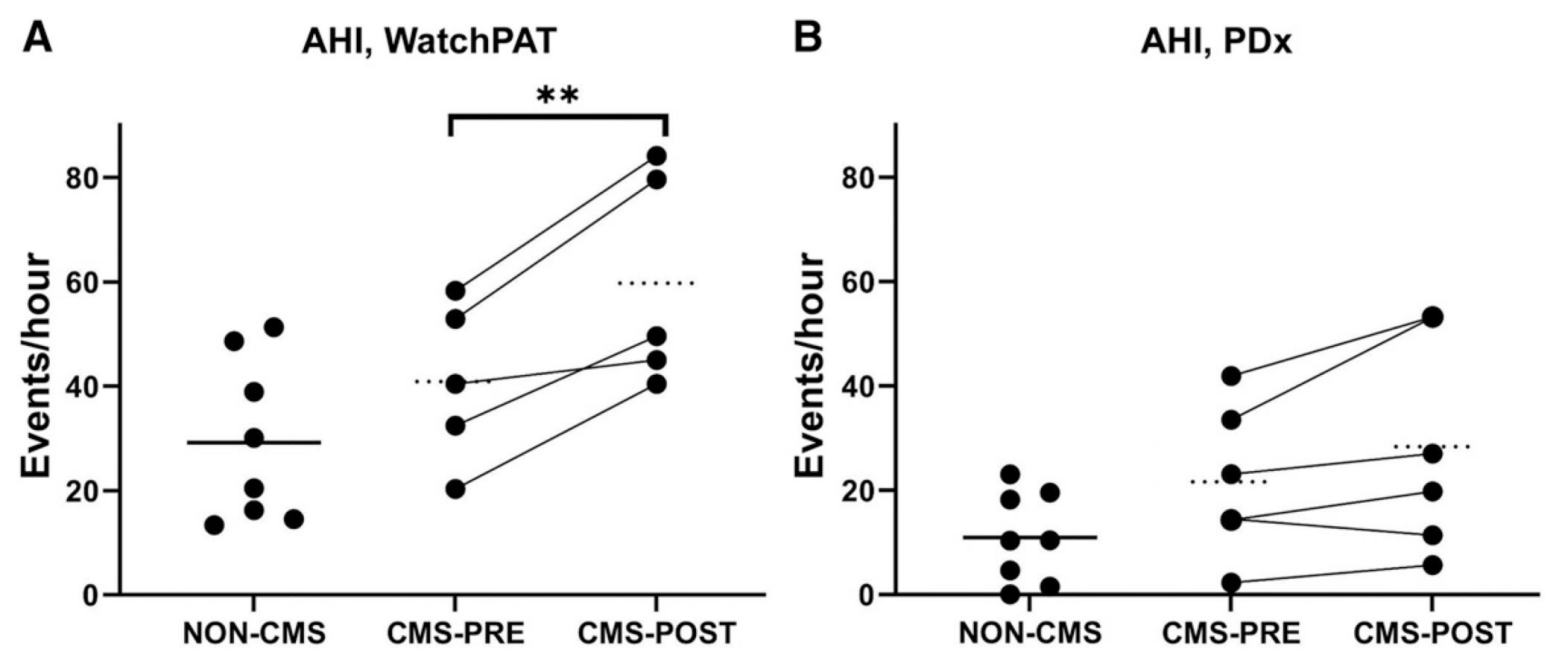
Effects of CMS presence, and IVHD in CMS, on sleep apnea severity. **(A)** AHI was not worse in CMS participants at baseline vs non-CMS controls, but IVHD worsened AHI in CMS participants, based on data from the WatchPAT device. **(B)** AHI was not higher in CMS participants vs non-CMS controls, and there was no significant effect of IVHD in CMS participants, using data from the PDx device. **P < .010. AHI = apnea-hypopnea index, CMS = chronic mountain sickness, IVHD = isovolumic hemodilution.

There was no difference in AHI between participants with and without CMS as measured by either device (Figure 1) (WatchPat: non-CMS 29.2 ± 5.5 vs CMS baseline 40.9 ± 6.9, *P* = .210; PDx: non-CMS 10.9 ± 3.1 vs CMS baseline 21.6 ± 5.9, *P* = .108). Similarly, there were no differences in NREM sleep AHI or REM sleep AHI between groups with either device (Table 2). However, central hypopneas were more frequent in CMS participants compared to non-CMS participants (4.2 ± 1.0 events/h vs 0.9 ± 0.4 events/h, *P* = .014).

IVHD in CMS participants increased AHI based on data from the WatchPAT device (40.9 ± 6.9 events/h to 61.5 ± 7.7 events/h, *P* = .009, Figure 1A). A similar trend was noted with the PDx device (21.6 ± 5.9 events/h to 28.4 ± 8.4 events/h, *P* = .086, Figure 1B). SDB became more severe after IVHD due primarily to an increase in AHI in NREM sleep (37.2 ± 6.4 events/h to 60.2 ± 8.9 events/h, *P* = .003, Table 2). IVHD did not significantly impact REM sleep AHI. There were no significant differences in respiratory event type (central or obstructive events) after IVHD in CMS participants. The increase in AHI after IVHD occurred primarily due to an increase in respiratory events in the supine position (supine AHI 38.7 ± 5.2 events/h before IVHD, and 59.2 ± 7.8 events/h after IVHD, *P* = .021, Table 2).

### IVHD worsens nocturnal oxygenation in CMS participants

**Figure 2:**
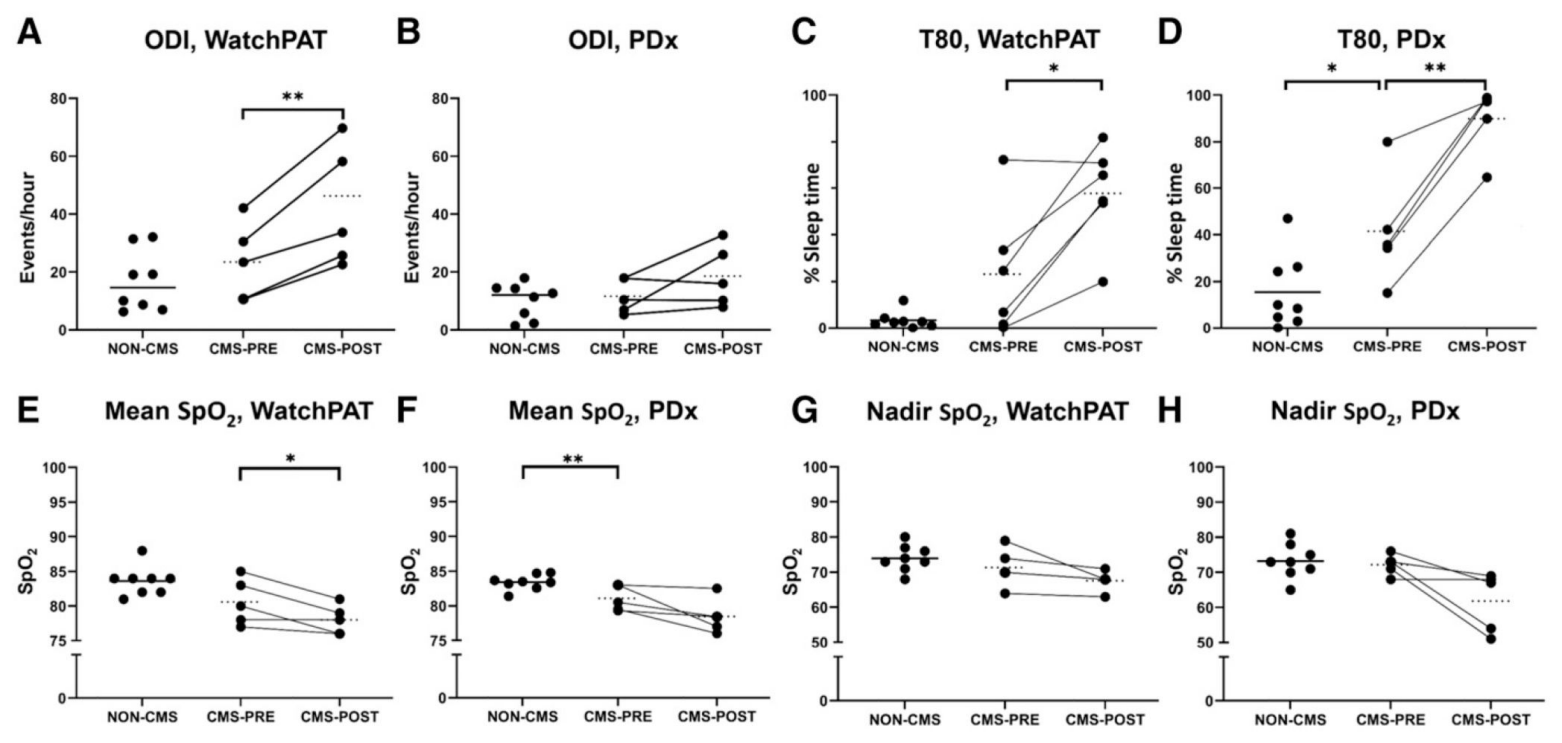
Effect of CMS presence, and IVHD in CMS, on nocturnal oxygenation. **(A,B)** The presence of CMS was not associated with any significant difference in ODI relative to those without CMS. However, based on data from the WatchPAT device, IVHD worsened ODI. **(C,D)** CMS presence did not worsen T80, but IVHD in those with CMS worsened T80 based on data from both devices. **(E,F)** CMS was associated with a reduction in mean nocturnal oxyhemoglobin saturation based on the PDx device but not the WatchPAT. However, IVHD in those with CMS worsened mean nocturnal saturation per the WatchPAT device. **(G,H)** CMS presence was not associated with any significant change in nadir oxyhemoglobin saturation, and IVHD did not impact nadir saturation based on either device. *P < .05, **P < .01. CMS = chronic mountain sickness, IVHD = isovolumic hemodilution, ODI = oxyhemoglobin desaturation index, T80 = percentage of sleep time with oxyhemoglobin saturation at or below 80%, SpO_2_ = peripheral oxyhemoglobin saturation.

There was no difference in oxyhemoglobin desaturation index (ODI, the number of desaturations of 3% or more per hour of sleep) between participants with and without CMS, when measured by either device (Figure 2A and Figure 2B). The T80 (sleep time spent with oxyhemoglobin saturation at or below 80%) was lower in those with CMS compared to those without CMS, based on data from the PDx device (15.44 ± 5.6% to 48.3 ± 11.1%, *P* = .0146), no difference were found with WatchPat device (Figure 2C and Figure 2D). Mean nocturnal saturation was similar between participants with and without CMS by the WatchPAT device (Figure 2E). However, data from the PDx device showed that mean nocturnal saturations were reduced in CMS participants relative to those without CMS (83.4 ± 0.4% to 80.6 ± 0.8, *P* = .005, Figure 2F). We found no changes in nadir oxyhemoglobin saturations with either device between participants with and without CMS (Figure 2G and Figure 2H).

Among those with CMS, IVHD doubled the ODI according to the WatchPAT device (23.5 ± 6.0 events/h to 46.2 ± 8.7 events/h, *P* = .008, Figure 2A). For the PDx device we found no significant difference before and after IVHD (11.7 ± 2.7 events/h, vs 18.6 ± 4.7 events/h, *P* = .177, Figure 2B). The T80 increased significantly after IVHD based on data from both devices (Figure 2C and Figure 2D) (WatchPAT: 23.2 ± 11.2% to 58.5 ± 10.6%, *P* = .012; PDx: 48.3 ± 11.1% to 89.8 ± 6.5%, *P* = .004). Mean nocturnal saturation worsened in CMS participants after IVHD based on the WatchPAT device (80.6 ± 1.5% to 78 ± 0.9%, *P* = .04, Figure 2E), but no significant differences were observed with the PDx device. We found no changes in nadir oxyhemoglobin saturations with either device after IVHD (Figure 2G and Figure 2H). However, there was a trend toward reduction in nadir saturation in CMS patients after IVHD based on the PDx device (72.2 ± 1.3 to 61.8 ± 3.8, *P* = .059, Figure 2H).

### Circulation time, heart rate, cardiac output, and acid-base status with IVHD

**Figure 3:**
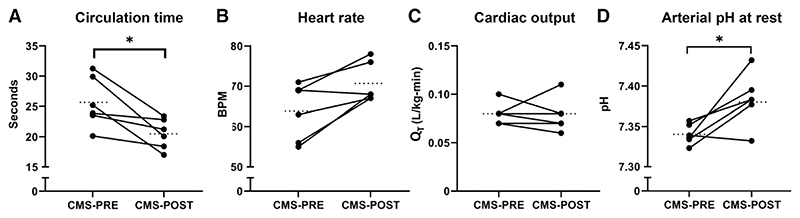
Effects of IVHD on circulation time, heart rate, cardiac output, and acid-base status. **(A)** IVHD in CMS participants reduced circulation time by 20.2% (25.7 ± 1.7 sec to 20.5 ± 1.0 sec, *P* = .022). **(B)** Mean nocturnal heart rate did not change after IVHD (63.8 ± 2.9 bpm to 70.7 ± 2.0 bpm, *P* = .062). **(C)** There was no difference in cardiac output normalized to body weight after IVHD (0.08 ± 0.00 L/min-kg to 0.07 ± 0.01 L/min-kg, *P* = .750). **(D)** Daytime arterial pH increased after IVHD (7.34 ± 0.005 to 7.38 ± 0.01, *P* = .026). *P < .05, **P < .01, ***P < .001. CMS = chronic mountain sickness, IVHD = isovolumic hemodilution.

We sought additional data in order to explain the pattern of generally worse oxygenation after IVHD in those with CMS. Specifically, we examined the effect of IVHD on cardiac output at wakeful rest, mean nocturnal heart rate, and circulation time, as markers of cardiac function, as well as changes in acid-base homeostasis. IVHD significantly reduced circulation time (20% reduction, *P* = .022, Figure 3A). There were no differences in mean nocturnal heart rate (Figure 3B) or cardiac output at rest (Figure 3C). Arterial pH increased after IVHD (7.34 ± 0.01 at baseline, vs 7.38 ± 0.01, *P* = .026, Figure 3D), corresponding to changes in base deficit (–10.2 ± 0.9 mEq/L at baseline, vs –7.0 ± 0.4, *P* = .029) but without significant changes in pCO_2_ (29.0 ± 1.6 at baseline vs 30.4 ± 0.9, *P* = .417).

### Objective sleep quality in CMS was unchanged with IVHD

**Figure 4:**
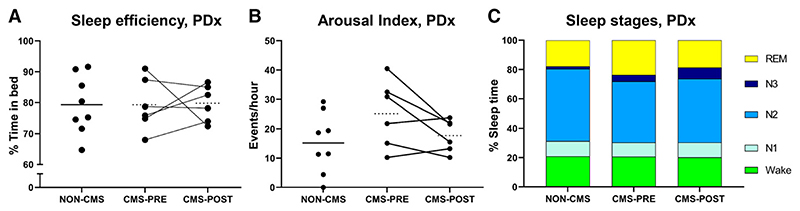
Effect of CMS presence and IVHD in CMS participants on sleep quality. **(A)** Sleep efficiency was not different in those with and without CMS, nor did IVHD impact sleep efficiency. **(B)** There were also no differences in arousal index in those with or without CMS, and IVHD did not impact arousal index. **(C)** Sleep stages were similar between groups as well. *P < .05, **P < .01, ***P < .001. CMS = chronic mountain sickness, IVHD = isovolumic hemodilution.

There were no significant differences in sleep efficiency, arousal index, or sleep staging in participants with or without CMS (Figure 4). Similarly, there were no differences in any parameter of sleep quality before and after IVHD. Only 3 of the CMS participants reported any sleep disturbance in the Qinghai questionnaire prior to IVHD (scores of 1, 1, and 3), and no participants reported any sleep disturbance at all (scores of 0 for all CMS participants) after IVHD.

### Comparison between 2 sleep devices

**Figure 5:**
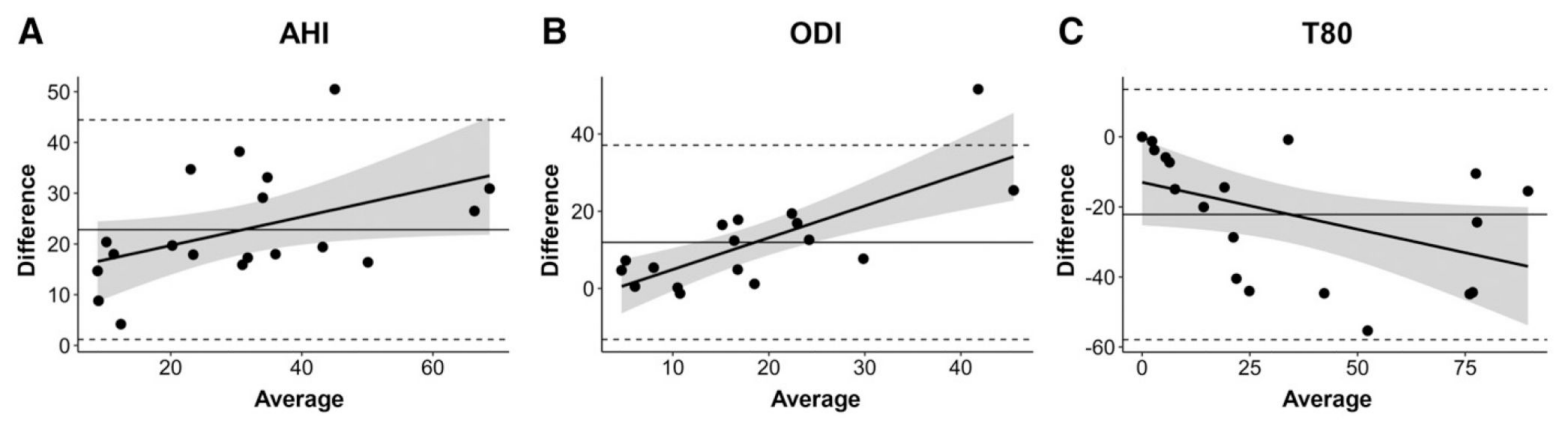
Bland-Altman comparison between 2 sleep devices (WatchPAT and PDx). **(A,B)** AHI and ODI measurements from the WatchPAT device were higher than those from the PDx device. The discrepancy between devices increased with increasing severity of each of these variables. **(C)** T80 values were lower based on data from the WatchPAT device, and again, the discrepancy between devices varied as a function of T80 severity. AHI = apnea-hypopnea index, ODI = oxyhemoglobin desaturation index, T80 = percentage of sleep time with oxyhemoglobin saturation at or below 80%.

**Figure 6:**
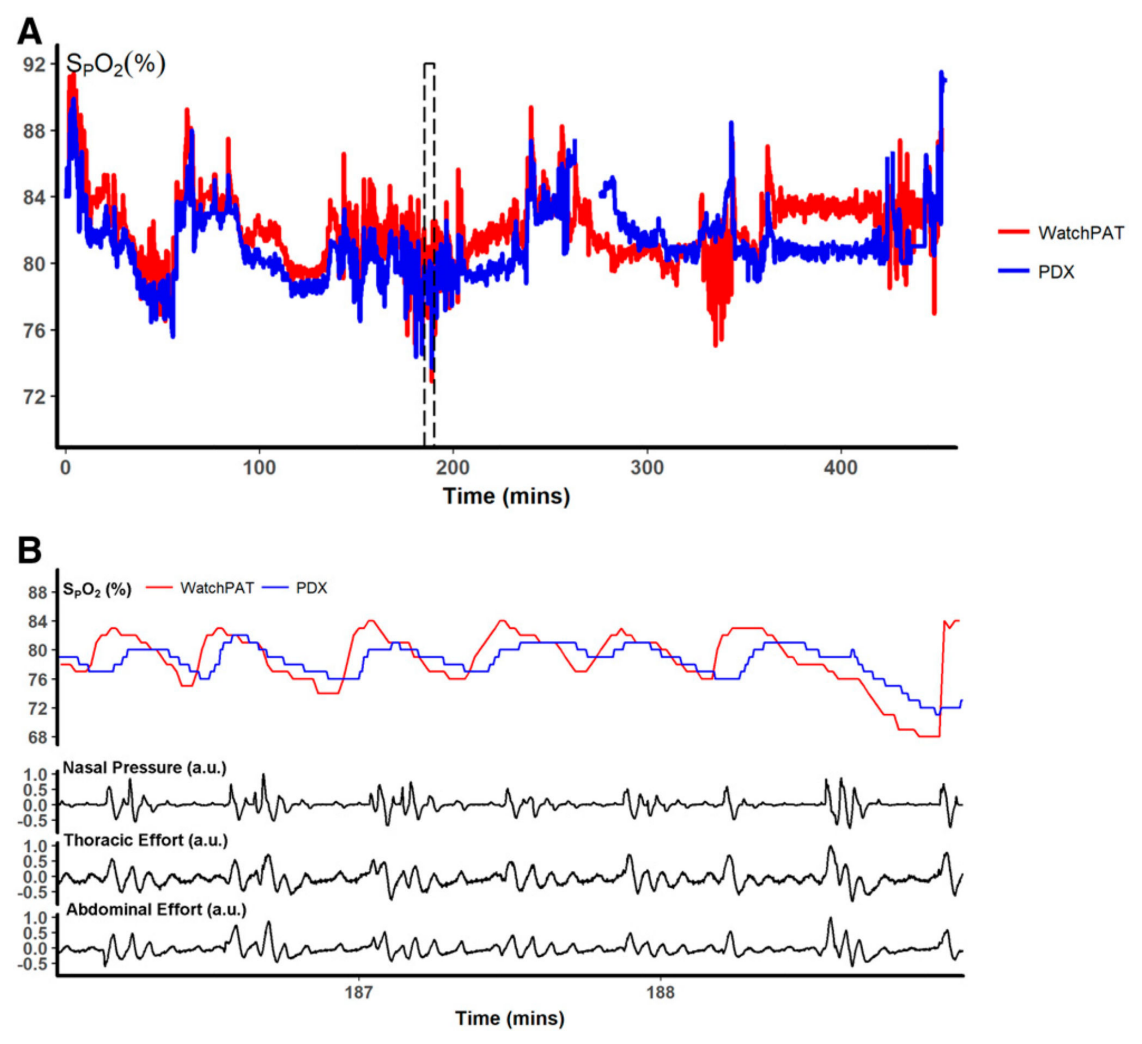
Oximetry tracings from 1 participant. **(A)** Whole-night oximetry tracings from the WatchPAT (red) and PDx (blue) devices. **(B)** Three-minute segment of oximetry (top), respiratory flow (middle), and respiratory effort (bottom 2). Respiratory flow and effort were obtained from the PDx device and the oximetry tracing from the WatchPAT device was synchronized to PDx for illustrative purposes. In general, there appear to be more exaggerated changes in saturation with the WatchPAT device. SpO_2_ = peripheral oxyhemoglobin saturation.

In several parameters of interest, we observed marked differences between the 2 sleep testing devices. Therefore, we used the Bland-Altman approach to determine whether these differences were a function of the severity of the outcome of interest. AHI was higher with measurements obtained from the WatchPAT device relative to the PDx device by a mean of 22.8 events/h (Figure 5A). For all the participants in the study (non-CMS participants, and every CMS participant both pre- and posthemodilution), values of AHI measurements with WatchPAT device were higher compared to those observed with PDx. Furthermore, the difference between devices was increased in participants with higher average AHI, indicating that more severe SDB was associated with a larger discrepancy between devices (β = 0.28, *P* = .048). ODI measurements were similarly discrepant, with higher values for the WatchPat device, by a mean of 11.9 events/h. Compared to the measurements performed with PDx, the differences between the 2 devices also varied as a function of ODI (Figure 5B). T80 readings were higher in the PDx device by 0.27% for each 1% increase in T80 (*P* = .050, Figure 5C). An example oximetry tracing during all night from 1 participant is shown in Figure 6A, with a detail showing the desaturation events observed during episodes of apnea/hypopnea in Figure 6B.

## Discussion

Chronic hypobaric hypoxia associated with high altitude poses several physiological challenges, including erythrocytosis, which, when excessive, is a fundamental feature of CMS. Some studies suggest that phlebotomy or hemodilution, each of which reduces polycythemia, improves oxygenation and reduces CMS symptoms.^21–24^ SDB is known to be highly prevalent at high altitude,^25^ but the effect of hemodilution on sleep apnea severity and sleep quality is relatively underexplored. Our findings suggest that IVHD may worsen SDB and nocturnal hypoxemia, at least acutely, but does not appear to affect sleep architecture.

In our study, we did not find that CMS was associated with significant increases in any measure of SDB severity: AHI was not higher in those with CMS vs non-CMS controls, and additionally, only few oxygenation metrics were different between groups. In some ways, this is unlike previous literature: Rexhaj et al noted markedly higher AHI and ODI in high-altitude dwellers with CMS vs those without CMS,^31^ and Villafuerte et al found that T80 was significantly higher in CMS patients compared to healthy highlanders.^29^ However, other studies either showed very low prevalence of SDB overall^27^ or did not observe that SDB was more severe in CMS.^28^ All of these studies, like our own, had relatively small sample sizes or were otherwise limited to young participants.^27^ We note that in our study, although SDB was not worse in CMS, the AHI, ODI, mean saturation, and T80 all trended worse in those with CMS.

In examining the effects of IVHD on SDB, we emphasize 2 major points. First, the change in AHI post-IVHD as measured by each device was driven primarily by an increase in the frequency of desaturations. Moreover, the arousal index actually trended lower following IVHD in CMS participants relative to baseline. Thus, IVHD was observed to worsen SDB severity independently of its effect on arousals.^41^ Second, worsening SDB after IVHD resulted from an increase in both central and obstructive events. This finding was somewhat surprising, since there appeared to be a larger absolute increase in NREM sleep–specific respiratory events than REM sleep–specific events, and central sleep apnea is far more prevalent in NREM sleep (vs REM).^42^

Our finding of generally poorer nocturnal oxygenation after IVHD in CMS participants also merits further consideration. Several metrics of nocturnal oxygenation worsened based on available data from at least 1 testing device: ODI and T80 were increased and mean nocturnal oxyhemoglobin saturation was reduced. It is not entirely clear why this occurred. Hemodilution has several physiological effects: Reducing red blood cell mass reduces blood viscosity; isovolemic, isotonic hemodilution in healthy volunteers has been shown to increase resting cardiac index,^43^ an effect we did not observe, and to improve cerebral perfusion, which might conceivably impact CMS symptoms. A reduction in blood viscosity could explain the reduction in circulation time we noted post-IVHD, but this result in isolation would not be expected to alter oxyhemoglobin saturation. The findings of reduced circulation time and no change in cardiac output after IVHD suggest that any worsening nocturnal oxygenation due to IVHD is not driven by poorer cardiac function, with the caveat that cardiac output was measured during day-time hours. Other possible explanations exist for our central findings. IVHD may reasonably have altered intravascular or extracellular fluid volume, despite efforts to minimize these effects in our experimental protocol. We did not measure the effect of IVHD on extracellular fluid volume, which may impact extravascular lung fluid and/or upper airway edema.

Differences in nocturnal oxygenation post-IVHD might instead revolve around changes in acid-base status. Central and peripheral respiratory chemoreflexes stimulating respiratory drive are elicited by elevated brain pCO_2_ (central), or activation of the carotid or aortic bodies (peripheral), the latter of which are triggered by hypoxemia, elevated pCO_2_, or reduced pH. The hypoxic response of peripheral chemoreceptors is also pH-dependent.^44^ Chemoreceptor stimulation results in arousal from sleep. Periodic breathing, as seen in many people at high altitude, may occur due to circulatory delay (elevated time constant of the central chemoreflex) or increased peripheral chemoreflex sensitivity.^44,45^ Thus, there are several reasons to consider acid-base homeostasis when examining our results. Serial nocturnal arterial pH measurements would inform this mechanistic explanation but were not performed in this study. However, if IVHD-induced changes to oxygenation were caused by acid-base homeostasis mediating a shift in the oxyhemoglobin saturation curve, this finding was not apparent in our daytime measurements of pH, in which participants became relatively alkalotic post-IVHD. As suggested above, relative metabolic alkalosis induced by IVHD would be predicted to have a minor depressant effect on respiratory drive.^46^ In theory, if drive to upper airway muscles were suppressed based on alkalemia, some worsening of SDB would be predicted. In any case, the specific mechanisms causing the effects we note on oxyhemoglobin saturations are not well understood.

Another finding from this study is that there are considerable differences between sleep testing devices in the outcomes we measured, particularly oxygenation. Recent data have demonstrated the limitations of pulse oximetry in a variety of clinical contexts^47–49^; SDB at high altitude may be characterized by hypoxemia which is worse than that observed at sea level, due to baseline sustained hypoxemia imposed by reduced atmospheric oxygen tension. In these environments, performance of US Food and Drug Administration–cleared pulse oximeters might be expected to vary, since performance testing is only done on healthy individuals who experience blood gas–verified arterial saturations between 70% and 100%.^50^ Since most of our participants had mean nocturnal saturations in the low 80s, and since at this baseline level of hypoxemia participants are often on the “steep” segment of the oxyhemoglobin saturation curve, we expect a large percentage of readings to fall outside the normal tested performance range of most commercial pulse oximeters. In addition to the potential for between-device variability in absolute oxyhemoglobin saturations, there also exists the possibility of differences in dynamic measures of oxygenation. In this cohort, differences in ODI between devices varied as a function of the frequency of events, suggesting that more rapid cycling was better detected by the WatchPAT device compared to the PDx device. Any observed differences could be magnified further by misclassification of individual events, since a saturation cutoff of 3% is typically used to define respiratory events, and since most respiratory events in our observed cohort were hypopneas (frequently being defined by changes in saturation) rather than apneas. We suggest that differences in absolute measure of saturation, and differences in the time constant of the sensors used, must be considered. These results demonstrate the lack of a comparative “gold standard” for measuring oxygenation (statically and dynamically) in this unique population. Nonetheless, we do note that despite these differences between devices, there was a clear trend of poorer oxygenation post-IVHD in CMS participants, suggesting that the overall effect of IVHD to worsen oxygenation is robust.

There are strengths and limitations to our study. First, participants were well characterized, and to our knowledge, this is the first report of the effects of IVHD on sleep in participants living at high altitude. However, the sample size in this study was quite limited, as was participant heterogeneity: All participants were adult males within a limited age range. Participants with and without CMS were also not age- or body mass index–matched, though there were no statistical differences between groups. With these limitations, definitive conclusions about the acute effects of IVHD are yet lacking. We also do not have gold standard testing for nocturnal oxygenation over time, such as repeated sampling of arterial blood, as this sort of invasive measure would have added considerable difficulty, expense, and risk. Moreover, we have limited understanding of the time course and clinical consequence of nocturnal hypoxemia in these participants. Specifically, it is unclear whether any observed changes on nocturnal oxygenation as a result of IVHD persist beyond 48 hours, and if not, whether short-term changes in nocturnal oxygenation are clinically relevant. Similarly, we also have limited insight into the mechanisms which might explain any changes in oxygenation after IVHD in highlanders with CMS.

In conclusion, this study demonstrates the effects of IVHD on sleep at high altitude in participants with and without CMS. The presence of CMS was not associated in our study with changes in sleep architecture or SDB severity. However, SDB and nocturnal oxygenation were worse in CMS participants 48 hours after IVHD. Future studies are needed to credential the findings in our study and to understand better the mechanisms which might account for these effects.

## Figures and Tables

**Table 1:** Baseline characteristics of study participants.

	Non-CMS (n = 8)	CMS-Pre (n = 6)	CMS-Post (n = 6)
Age, years	52.1 ± 3.1	49.7 ± 5.7	—
Body weight, kg	64.3 ± 2.3	69.6 ± 6.4	67.2 ± 6.1**
BMI, kg/m^2^	24.8 ± 2.4	25.2 ± 4.5	24.3 ± 1.7**
[Hb], g/dl	17.8 ± 0.5	22.2 ± 0.6###	17.7 ± 0.2***
CMS Score	1.1 ± 1.0	10.0 ± 5.5##	1.2 ± 1.2*

Values expressed as mean ± SEM.

## *P* < .01,

### *P* < .001 non-CMS vs CMS-pre.

* *P* < .05,

** *P* < .01,

*** *P* < .001 CMS-pre vs CMS-post.

BMI = body mass index, CMS = chronic mountain sickness, Hb = hemoglobin, SEM = standard error of the mean.

**Table 2:** Characteristics of respiratory events with WatchPat and PDx devices.

	PDx			WatchPat		
	Non-CMS	CMS-Pre	CMS-Post	Non-CMS	CMS-Pre	CMS-Post
REM sleep AHI	18.0 ± 5.7	22.3 ± 4.6	24.9 ± 7.6	41.9 ± 4.8	50.5 ± 7.7	63.0 ± 5.4
NREM sleep AHI	9.3 ± 3.1	21.4 ± 6.4	30.2 ± 9.5	25.4 ± 6.2	37.2 ± 6.4	60.2 ± 8.9**
Supine AHI	—	—	—	33.0 ± 7.2	38.7 ± 5.2	59.2 ± 7.8*
Nonsupine AHI	—	—	—	33.2 ± 17.0	42.5 ± 6.5	59.8 ± 7.7
Central apnea index	2.6 ± 1.4	3.5 ± 2.5	5.7 ± 2.9	—	—	—
Obstructive apnea index	1.6 ± 0.8	4.0 ± 1.5	5.0 ± 3.1	—	—	—
Central hypopnea index	0.9 ± 0.4	4.2 ± 1.0#	5.9 ± 1.3	—	—	—
Obstructive hypopnea index	6.2 ± 1.9	10.0 ± 3.3	11.4 ± 3.9			

Values expressed as mean ± SEM, events/h.

# *P* < .05, Non-CMS vs CMS-pre.

* *P* < .05, CMS-pre vs CMS-post.

** *P* < .01, CMS-pre vs CMS-post.

AHI = apnea-hypopnea index, NREM = non–rapid eye movement, REM = rapid eye movement, SEM = standard error of the mean.

## Brief Summary

### Current Knowledge/Study Rationale

Chronic mountain sickness (CMS) is common among Andean highlanders and is characterized by excessive erythrocytosis and sleep disturbance, among other manifestations. Although phlebotomy is commonly performed in patients with CMS, there are no data regarding the effect of phlebotomy or hemodilution on sleep quality or sleep-disordered breathing severity.

### Study Impact

In this study, the presence of CMS was not associated with changes in sleep architecture or sleep-disordered breathing severity; however, sleep-disordered breathing and nocturnal oxygenation were worse in CMS participants 48 hours after isovolemic hemodilution. The role of phlebotomy or hemodilution in those with CMS merits further, larger scale, study.

## Abbreviations

AHI: apnea-hypopnea index
CMS: chronic mountain sickness
IVHD: isovolumic hemodilution
NREM: non–rapid eye movement
ODI: oxyhemoglobin desaturation index
REM: rapid eye movement
SDB: sleep-disordered breathing
T80: percentage of sleep time with oxyhemoglobin saturation at or below 80%
